# A 12-Year Review of Upper Extremity Deep Vein Thrombosis—Are They the Same as Lower Extremity Deep Vein Thrombosis?

**DOI:** 10.3390/jcm13216440

**Published:** 2024-10-27

**Authors:** Patrick Leung, Brandon Lui, Julie Wang, Prahlad Ho, Hui Yin Lim

**Affiliations:** 1 Department of Haematology, Northern Hospital, Epping, VIC 3076, Australiahuiyin.lim@nh.org.au (H.Y.L.); 2 Northern Clinical Diagnostics and Thrombovascular Research (NECTAR) Centre, Northern Health, Epping, VIC 3076, Australia; 3 Department of Medicine (Northern Health), University of Melbourne, Epping, VIC 3076, Australia

**Keywords:** deep vein thrombosis, upper extremity, lower extremity, Paget–Schroetter syndrome, bleeding, recurrence

## Abstract

**Background:** Upper extremity deep vein thrombosis (UEDVT) is uncommon but not insignificant. The current literature is limited, and the management is largely extrapolated from the treatment of lower extremity DVTs (LEDVT). **Methods**: A retrospective review was conducted on patients diagnosed with UEDVT at Northern Health, Victoria, Australia, between December 2010 and December 2022. Medical records were reviewed to assess baseline characteristics and treatment outcomes. The results were compared to our previously collected data for LEDVTs. **Results**: 137 patients with UEDVT were identified (52.6% females; median age 62 years, IQR 46–74 years). A total of 105 patients (76.6%) had at least one provoking factor at the time of diagnosis, most commonly malignancy (45.7%) and/or indwelling venous devices (58.1%). Fourteen patients (10.1%) were subsequently diagnosed with Paget–Schroetter syndrome, with nine receiving endovascular or surgical intervention. A total of 109 patients (79.6%) received limited therapeutic anticoagulation (median 3 months, IQR 1.5–6.0 months) with enoxaparin, the most common anticoagulant used. Six patients had major bleeding (5.2/100-patient-years), and seven developed clot progression while on anticoagulation (6.0/100-patient-years). Ten patients had recurrent VTE following anticoagulation cessation (4.6/100-patient-years). There were no significant differences seen in the complication rate between catheter-related UEDVT and other UEDVTs. Compared to LEDVT, UEDVT was more likely provoked with comparable complication rates. **Conclusions**: UEDVTs were commonly associated with a provoking factor, with indwelling catheters and/or malignancies being the most common. Interestingly, catheter-related UEDVT had comparable clot progression/recurrence and major bleeding compared to other UEDVTs and LEDVTs, which may be confounded by relatively high rates of malignancy.

## 1. Introduction

Upper extremity deep vein thrombosis (UEDVT) accounts for approximately 5% of all deep vein thromboses (DVTs) [1,2]. They can be defined as DVTs occurring in any vein of the upper extremity or thoracic inlet, including the radial, ulnar, brachial, axillary, subclavian, brachiocephalic, and jugular veins [3]. UEDVTs can be categorised as either primary or secondary. Primary UEDVT, or Paget–Schroetter syndrome (PSS), is a rare thrombotic manifestation of thoracic outlet syndrome, with an estimated annual incidence of 2 per 100,000 individuals [4]. Repetitive compression of the subclavian vein between the clavicle and first rib leads to vessel wall scarring and disrupted blood flow, predisposing to thrombosis formation [5]. Secondary UEDVTs are provoked, commonly in the setting of intravascular devices, such as central venous catheters and implantable cardiac devices, or malignancy [6,7,8]. Secondary UEDVTs are far more common than PSS, forming up to 80–90% of cases [9,10]. 

Clinical suspicion of UEDVT requires confirmation with imaging for diagnosis. While computer tomography (CT) venography is the gold standard for the diagnosis of UEDVT [11], the invasive nature and contrast exposure make it a less desirable first-line imaging modality. As such, ultrasonography is often preferred due to its relative availability and inexpensiveness. Timely diagnosis of UEDVT is important in order to prevent morbidity and mortality. In a systematic review of studies examining UEDVTs, post-thrombotic syndrome (PTS) has been reported in 7–46% of patients [12]. Concurrent symptomatic pulmonary embolism (PE) has also been noted with UEDVT, occurring in approximately 5% of patients [13,14], though asymptomatic PEs may also be present in a considerable proportion of patients [15].

The management of UEDVTs is largely extrapolated from the treatment of lower extremity DVTs (LEDVTs). The backbone of treatment is anticoagulation, increasingly with direct oral anticoagulants (DOACs), though parenteral anticoagulants and warfarin are also used. Interventional techniques, such as catheter-directed thrombolysis (CDT) and surgical decompression of the thoracic outlet via a first rib resection (FRR), have been used in the setting of PSS, with good functional outcomes and prevention of recurrence [16,17]. However, current evidence for UEDVT management is limited, and there are no widely established guidelines. Questions remain regarding whether all UEDVT should receive anticoagulation, with the most recent American College of Chest Physician guidelines only recommending anticoagulation in axillary veins or more proximal UEDVTs [18]. Furthermore, the optimal duration in non-catheter-provoked UEDVT or PSS is not well established. Surgical guidelines are also lacking, in particular with respect to the utility of catheter-directed thrombolysis and the timing of surgical decompression [19]. 

In this retrospective study, we examined the epidemiology and treatment outcomes of UEDVTs at our institution. We also compared outcomes to those observed in patients with LEDVTs.

## 2. Materials and Methods

Patients with UEDVT were identified via medical coding between December 2010 and December 2022 at the Northern Hospital in Melbourne, Australia. The diagnosis was confirmed by a review of medical records and radiology imaging reports. UEDVT was defined as thrombosis involving the radial, ulnar, brachial, axillary, subclavian, brachiocephalic, and jugular veins. Patients with isolated superficial venous thrombosis were excluded. Baseline demographic details, provoking factors of UEDVT, treatment, and outcomes were recorded. An event was considered provoked if an identifiable provoking factor was present within 8 weeks of the VTE presentation. Complications were also noted, including recurrent or breakthrough thrombosis and bleeding. Major bleeding was defined as per the International Society of Thrombosis and Haemostasis (ISTH) guidelines [20,21]. The length of the follow-up was defined as the date the patient was last reviewed at our network in either inpatient or outpatient settings. LEDVT data used (*n* = 2168, 51.8% females, median age 65 years, IQR 49.0–77.0) were from a retrospective study performed by our research group over the course of 10 years (January 2011–December 2020) [22].

Statistical analysis was performed on Microsoft Excel, IBM SPSS, version 22.0 (SPSS Inc., Chicago, IL, USA) and Stata version 18.0 (StataCorp, College Stations, TX, USA). Comparisons were made between UEDVT and LEDVT populations with respect to risk factors, anticoagulation choice, and duration and complications. Categorical variables were analysed using the chi-squared or Fisher’s exact test, depending on the sample size. Statistical significance was set at *p* < 0.05. Time-to-event analysis was conducted with major bleeding during therapeutic anticoagulation, clot progression during therapeutic anticoagulation, and clot recurrence after anticoagulation cessation as endpoints, expressed as events per 100 patient-years (100 PY). Patients were censored at the date of death or last known follow-up. For major bleeding and clot progression, a comparison of events between groups was conducted using univariate cox proportional hazards regression. For clot recurrence, competing-risk regression using death as a competing risk was performed using the method by Fine and Gray.

## 3. Results

### 3.1. Demographics and Baseline Characteristics

A total of 137 patients were identified, with a median age of 62 years (IQR 46–74 years; 65 males, 72 females). The majority of cases were identified on ultrasonography (125 patients, 91.2%). The majority of the patients had multiple vein involvement (*n* = 72, 52.6%). The most commonly affected venous tributaries were the jugular veins (*n* = 66), including 23 patients with internal jugular vein DVT alone, followed by subclavian vein involvement (*n* = 61), including 15 patients with isolated subclavian DVT. Fifty-eight patients had brachial vein involvement, including 17 patients with brachial vein involvement only, and 32 patients reported axillary vein involvement, with 10 patients having isolated axillary vein DVT. 

Nearly all patients were symptomatic at the time of diagnosis (93.4%). Ten patients (7.2%) were concurrently diagnosed with PE. Six patients were on therapeutic anticoagulation, and 25 patients were on prophylactic anticoagulation (as VTE prophylaxis while an inpatient) at the time of diagnosis. Demographics and provoking factors are shown in Table 1. 

One-hundred-and-five patients (76.6%) had at least one documented provoking factor (Table 1). Seventeen patients (12.4%) were also noted to have had a prior VTE, none of which were UEDVTs. The most common provoking factor was indwelling venous devices (*n* = 61, 58.1% of provoking factors), including 6 patients with implantable cardiac devices and 27 with concurrent malignancy. Of the 55 patients with a venous catheter, approximately half had the line removed prior to or at the time of diagnosis (31/55; 56.4%); three patients died with their catheter in situ. The remaining 21 patients had their lines removed at a median of 3 days (IQR 1–14 days) following diagnosis. A total of 48 patients (45.7%) had active cancer at the time of UEDVT. Twenty-six patients were receiving chemotherapy at the time of diagnosis; 21/26 (80.8%) had an indwelling venous catheter. Of the 34 patients with unprovoked UEDVT, 11 patients (8.0% overall) were retrospectively diagnosed with Paget–Schroetter syndrome (PSS), which is typically effort-induced and associated with the compression of the subclavian vein at the thoracic outlet.

Sixty-one patients underwent thrombophilia testing (including factor V Leiden mutation, prothrombin gene mutation, antithrombin levels, protein C and S, lupus anticoagulant, anti-cardiolipin and β2-glycoprotein antibodies), with six patients identified as factor V Leiden heterozygous. Other testing did not yield any positive results when tested appropriately.

### 3.2. Management with Anticoagulation

Anticoagulants used in treatment are shown in Table 1. A total of 132 patients (96.4%) received therapeutic anticoagulation for initial management—low-molecular-weight heparin (LMWH) was the most common agent used (93/132; 70.5%). Four patients received only prophylactic anticoagulation; two patients had concurrent thrombocytopenia; one had active bleeding, and one was concurrently diagnosed with atrial fibrillation and met the criteria for prophylactic apixaban. One patient did not receive any anticoagulation due to a prior bleeding history. Direct oral anticoagulants (DOACs) were the most commonly used subsequent anticoagulation following the initial treatment (53/132; 40.2%), followed by LMWHs (36.4%) and warfarin (22.7%). Anticoagulation was not continued in six patients, including two patients with malignancy who died, two patients who had the involved catheter removed, and one due to bleeding/disseminated intravascular coagulopathy.

### 3.3. Management in Patients with PSS

Patients diagnosed with PSS were significantly younger than those with provoked UEDVT (median age 32.5 years, IQR 19.5–46.5 years vs. 65 years, IQR 49–75 years; *p* < 0.001). There was no significant difference in gender (*p* = 0.347). Of the 14 patients diagnosed with PSS, 9 (64.3%) underwent surgical intervention (Table 2). A range of procedures were utilised, most commonly pharmacomechanical thrombectomy with angioplasty and first rib resection (FRR). One case was treated with thrombectomy, and the first rib resection was complicated by a post-procedural PE. There were no other significant complications were noted. The remaining five patients were treated with therapeutic anticoagulation only—there were no instances of clot recurrence, major bleeding, or symptoms suggestive of PTS.

### 3.4. Follow-Up and Complications

A total of 116 patients (85.7%) received ongoing follow-up at our institution (median 21 months; IQR 4–50 months), while 13 patients died acutely during the same inpatient admission. Eight patients (5.8%) had their care transferred elsewhere or did not attend follow-up appointments. The median duration of the acute phase (therapeutic) treatment was 5.0 months (IQR 2.0–8.4 months; 95% confidence interval (CI) 4.9–5.1). If a stop date of anticoagulation was identified, the treatment duration was censored at this time. If a decision was made for indefinite anticoagulation or was lost to the follow-up, the treatment duration was censored at the date of the follow-up. A total of 109 patients (79.6%) received a limited course of anticoagulation (median 3 months; IQR 1.5–6.0 months), while 10 received indefinite anticoagulation. Seventeen patients had an unknown duration, either due to a lack of follow-up or unclear documentation. One patient was still receiving ongoing treatment at the time of the last review, with the duration yet to be finalised. Five patients had documented symptoms suggestive of PTS at their last review.

Repeat imaging was also performed in 85 patients (median time to imaging 89 days; IQR 46.0–138.0 days) (62.8%). A total of 78 patients underwent repeated US only; three patients had US in conjunction with a CT venogram, two had US with a ventilation-perfusion scan, and two had a CT venogram alone. The results are summarised in Table 3. The majority of patients (*n* = 77, 90.6%) had no residual clot or reduced clot burden. In patients with diagnosed PSS, 13 patients had repeat imaging, all of which either had a reduced or no residual clot burden (median 71 days to repeat imaging; IQR 45.0–118.0 days). 

Six patients had major bleeding whilst on anticoagulation medication (5.15/100 PY), including four patients within 60 days of commencing anticoagulation treatment (median time to bleed 24.5 days, IQR 3.8–180.0 days). Two patients were on warfarin (one was supratherapeutic), three patients were on therapeutic enoxaparin, and one was on an intermediate dose of unfractionated heparin. While there was a trend towards an increased rate of major bleeding in patients with concurrent malignancy (Hazard ratio (HR) 4.49, 95%CI 0.81–24.88, *p* = 0.086), there was no significant difference in the rate of major bleeding across the subgroups (catheter-related 5.3/100 PY vs. other provoked 13.5/100 PY vs. unprovoked 2.2/100 PY) (Table 4).

Seven patients developed clot progression while on therapeutic anticoagulation (6.0/100 PY). This included two patients with active malignancy, three patients with venous catheters, one patient with both malignancy and a venous catheter, and one patient who was diagnosed with PSS. Additionally, ten patients had recurrent VTE post anticoagulation cessation (4.5/100 PY, median time to event 10 months, IQR 1.8–38.8 months; Table 5), including three patients with malignancies. Seven of these patients had unprovoked recurrent VTEs. When comparing subgroups, there was no significant difference in clot progression or recurrence between patients with catheter-related UEDVT compared to those with non-catheter-provoked or unprovoked UEDVT. There was a total of 19 deaths during the course of the follow-up—one was partially attributed to clot progression and the development of PE. No deaths secondary to major bleeding occurred.

### 3.5. Comparison to LEDVT

Table 6 summarises the comparisons between UEDVT and LEDVT cases. LEDVT WAS more likely to be symptomatic (97.8% vs. 92.8%, *p* < 0.001), while more UEDVTS were provoked (82.6% vs. 60.7%, *p* < 0.001), with malignancy and catheters being the primary risk factors (*p* < 0.001). There were also significant differences in THE anticoagulation of choice, with LEDVT more commonly treated upfront with DOACs (25.7% vs. 14.5%, *p* < 0.001) and warfarin in UEDVT (5.8% vs. 1.2%, *p* = 0.001). There was more ongoing enoxaparin use in UEDVT and warfarin use in LEDVT (*p* < 0.001). The median duration of anticoagulation was longer in LEDVT patients (5 months vs. 3 months, *p* = 0.008). There were no significant differences in complications when comparing patients with UEDVT to those with LEDVT (Table 6).

## 4. Discussion

This retrospective audit of UEDVTs at our institution highlights the demographics and risk factors of these patients, as well as our experiences with the management of this condition. Interestingly, catheter-related UEDVT appears to have a comparable complication rate to non-catheter-related UEDVT. We also compared these findings with previously collected data on LEDVTs [22] to explore whether UEDVTs can be considered analogous and treated similarly. We found that UEDVT were more likely to be provoked with malignancy and venous catheters as predominant risk factors, although there were no significant differences in complication rates compared to LEDVT.

Consistent with reports in the literature [6,7,8], we found that indwelling venous devices (including venous catheters and implanted cardiac devices) and malignancy were the major provoking factors for UEDVT. The incidence of catheter-related UEDVT has been reported as high as 15% in large systematic reviews, with additional risk factors of malignancy and critical illness representing the highest risk cohorts [23,24]. The incidence of UEDVT secondary to transvenous leads from implanted cardiac devices is comparatively less common (~0.5%) [25]. Notably, PSS was responsible for approximately 10% of UEDVT in our patient population, higher than what was reported in the RIETE registry of 5% [26]. Overall, the rate of unprovoked events was significantly lower compared to LEDVT (24.8% vs. 39.3%), highlighting the need to consider secondary causes as well as PSS, particularly in younger patients.

There were no significant differences in the major bleeding and clot progression or recurrence rates when comparing catheter-provoked to non-catheter-provoked UEDVT and unprovoked UEDVT. This may be, in part, due to a relatively increased rate of malignancy in this cohort of patients, who are known to be at increased risk of bleeding and VTE [27]. Although small numbers were present, our study reported a trend towards increased risk of major bleeding in patients with malignancy but comparable rates of clot progression or recurrence. Of note, the RIETE registry also reported an increased risk of major bleeding and recurrent PE in catheter-related UEDVT compared to its unprovoked counterpart, with no increased clot recurrence in non-catheter-provoked UEDVT [1].

Despite the paucity of consensus guidelines in the literature, surgical intervention appears to be the preferred management of primary UEDVT. Current evidence suggests catheter-directed thrombolysis (CDT) and FRR as the optimal method of management. In a retrospective case series, Hoexum et al. examined 91 patients treated with PSS [17]. The 21 patients treated with CDT and FRR demonstrated significantly better functional outcomes and were less likely to have PTS when compared to those managed only with anticoagulation. The authors also noted minimal complications secondary to surgical intervention. However, in a single-site case series of 26 patients treated for PSS, 3 patients re-occluded their subclavian vein after CDT and FRR but remained asymptomatic [28]. The authors postulated that this may have been secondary to the formation of collaterals, and thus, the benefit of decompressive surgery may be questionable in this subgroup of PSS patients. In our study, a wide range of techniques were employed, including pharmacomechanical thrombectomy techniques, which have been demonstrated to result in higher rates of a significant reduction in clot burden, as well as shorter intervention time, which may prevent adverse events [29,30]. Conversely, several patients were treated conservatively with therapeutic anticoagulation only. No adverse events were reported with this approach, nor were there any documented symptoms of PTS; specific scores of function were not assessed, however. While limited by the small sample size, therapeutic anticoagulation may represent a reasonable alternative if invasive procedures are unsafe or undesired.

The current management of UEDVT is largely based on experience with LEDVT, and aside from catheter-related DVTs, there are no unifying guidelines. The anticoagulation choice appeared to vary between the two groups; enoxaparin was favoured initially in UEDVT and DOACs with LEDVT, with a larger proportion of the former continuing on enoxaparin, which may reflect the higher rates of cancer patients. This audit occurred between 2010 and 2022, with warfarin and/or enoxaparin as the dominant options in the first third of the decade, followed by a period of transition to the now predominant DOAC use. In this study, there were no significant differences in the major bleeding and clot progression/recurrence rate between both cohorts, similar to other studies [1,2], although there was a trend towards more clot progression and major bleeding in the UEDVT cohort. This may be attributable to the heightened risk of bleeding and thrombosis in patients with malignancy. In the RIETE registry, Cote et al. observed that patients with non-catheter-provoked UEDVT (50% had malignancy listed as a risk factor) had similar rates of DVT and PE recurrence compared to provoked LEDVT during anticoagulation [1]. This sentiment was echoed in the analysis of the GARFIELD-VTE registry [2], in which similar rates of recurrent VTE were observed across UEDVT and LEDVT with no difference in major bleeding. Of note, the ARM-DVT (NCT02945280) is a current prospective study examining the safety and effectiveness of apixaban in the treatment of UEDVT, with study completion expected by the end of 2023.

We acknowledge the relatively small sample size of this cohort. The retrospective nature of this study is subject to treatment selection biases, and given the small number of adverse events, this study was not powered to investigate treatment efficacy. There was a lost to follow-up rate of about 15.2%, which may impact the duration of anticoagulation and adverse events. A significant proportion of the patients were managed with enoxaparin and/or warfarin, consistent with the standard of care in the earlier years of the audit. Nevertheless, our study identified the risk factors and outcomes of treated UEDVT, including those with PSS.

## 5. Conclusions

In summary, this retrospective review highlights our experiences with UEDVT. Malignancy and catheters were the most common provoking factors, which is in line with observations in the literature. A total of 10% of events were secondary to PSS, with nearly two-thirds successfully managed with surgical intervention. The high rates of provoking events suggest the need for the careful evaluation of risk factors in UEDVT. The trend towards major bleeding in patients with concurrent malignancy suggests that individualised patient management may be required in this cohort to balance bleeding and thrombosis risk. Interestingly, catheter-related UEDVT has a comparable rate of complications compared to other non-catheter-related-provoked and unprovoked UEDVT. There were also no significant differences in complication rates between UEDVT and LEDVT. These findings may be influenced by the higher rates of concurrent malignancy among UEDVT patients in our study.

## Figures and Tables

**Table 1:** Baseline demographics and summary of provoking factors in patients with UEDVT.

	Catheter-Related UEDVT	Non-Catheter-Related-Provoked UEDVT	Unprovoked UEDVT
Patients, N	61	44	32
Age, median (IQR)	65 (50.0–74.0)	69 (46.8–75.8)	49.5 (31.5–66.3)
Female, N (%)	35 (57.4)	24 (54.5)	13 (40.6)
Prior VTE	0	7 (15.9)	3 (9.4)
**Provoking factors, N (%)**			
Malignancy	27 (44.3)	21 (47.7)	0
Recent surgery	18 (29.5)	11 (25.0)	0
Injury/prolonged immobility	15 (24.6)	16 (36.4)	0
HRT/OCP	1 (1.6)	5 (11.4)	0
Factor V Leiden heterozygous	2 (3.3)	2 (4.5)	2 (6.3)
Retrospective diagnosis of PSS	0	3 (6.8)	11 (34.4)
**Initial anticoagulation, N (%)**			
Heparin	2 (3.3)	5 (11.4)	4 (12.5)
LMWH	48 (78.7)	33 (75.0)	20 (62.5)
DOAC	8 (31.1)	5 (11.4)	7 (21.9)
Prophylaxis *	2 (3.3)	1 (2.3)	1 (3.1)
Not given	1 (1.6)	0	0
**Ongoing anticoagulation, N (%)**			
LMWH	24 (39.3)	22 (50.0)	2 (6.25)
Warfarin	14 (23.0)	6 (13.6)	10 (31.3)
DOAC	20 (32.8)	13 (29.5)	20 (62.5)
No	3 (4.9)	3 (6.8)	0

Initial and subsequent anticoagulation is also shown. Abbreviations: HRT—hormonal replacement therapy; OCP—oral contraceptive pill; PSS—Paget–Schroetter syndrome; LMWH—low molecular weight heparin; DOAC—direct oral anticoagulants. * Prophylactic agents used included heparin, LMWH and apixaban.

**Table 2:** Summary of interventions in patients diagnosed with PSS.

Surgical Intervention	Number of Patients (n)
Pharmacomechanical thrombectomy + angioplasty + FRR	4
Pharmacomechanical thrombectomy + angioplasty	1
FRR + angioplasty	1
Thrombectomy + FRR	1
FRR alone	1
CDT + angioplasty	1

Abbreviations: FRR—first rib resection; CDT catheter-directed thrombolysis.

**Table 3:** Summary of patients who had repeat imaging and their findings.

**Imaging < 90 days**	43 (median 46 days; IQR 26.5–71.5 days)
No residual deep vein thrombosis	19 (44.2%)
Reduced clot burden	19 (44.2%)
Stable	4 (9.3%)
Extension	1 (2.3%)
**Imaging ≥ 90 days**	42 (median 145 days; IQR 118–209.8 days)
No residual deep vein thrombosis	19 (45.2%)
Reduced clot burden	20 (47.6%)
Stable	2 (4.8%)
Unknown	1 (2.4%) *

* Externally reported scan where no comparison to initial imaging could be made.

**Table 4:** Complications by subgroup.

	Major Bleeding, n (Events/100 Patient-Years)	Clot Progression, n (Events/100 Patient-Years)	Clot Recurrence, n (Events/100 Patient-Years)
**Overall**	6 (5.15)	7 (6.00)	10 (4.55)
No malignancy	2 (2.35)	4 (4.70)	6 (3.80)
With malignancy	4 (12.71)	3 (9.53)	4 (6.43)
HR (95% CI) *	4.49 (0.81–24.88) *p* = 0.09	1.39 (0.31–6.21) *p* = 0.67	0.87 (0.25–3.08) *p* = 0.83
**Catheter-related**	3 (5.27)	4 (7.03)	6 (5.24)
With malignancy	2 (7.83)	1 (3.91)	4 (8.01)
No malignancy	1 (3.19)	3 (9.58)	2 (3.09)
**Provoked, non-catheter**	2 (13.46)	2 (13.46)	2 (3.69)
HR (95% CI) **	1.38 (0.22–8.58) *p* = 0.73	0.80 (0.15–4.37) *p* = 0.80	0.44 (0.10–2.09) *p* = 0.30
**Unprovoked**	1 (2.23)	1 (2.23)	3 (5.83)
HR (95% CI) **	0.45 (0.05–4.40) *p* = 0.49	0.43 (0.05–3.89) *p* = 0.46	0.98 (0.20–4.79) *p* = 0.98

Abbreviations: HR—hazard ratio; CI—confidence interval. * HR (Hazard ratio) compared against patients without malignancy. ** HRs compared against catheter-related events. For clot recurrence, competing risk of death has been accounted for.

**Table 5:** Summary of patients with recurrent VTE after initial UEDVT diagnosis.

Initial UEDVT	Recurrent	Months off Anticoagulation
Catheter-provoked	Unprovoked LEDVT	4
Malignancy	Unprovoked PE	8
Unprovoked	Unprovoked UEDVT	1
Injury (trauma)	Unprovoked LEDVT	62
Malignancy/catheter	Unprovoked UEDVT	45
Injury	Unprovoked PE	20
Catheter-provoked	Unprovoked PE	1
Malignancy/catheter	Catheter-associated SVT	12
Unprovoked	Postoperative PE	0.5
Catheter-provoked	Malignancy associated PE	60

**Table 6:** Comparison of risk factors, management and complications between UEDVT and LEDVTs.

	LEDVT	UEDVT	*p*-Value
Cases	2168	137	-
Age, median (IQR)	65.0 (49.0–77.0)	62.0 (46.0–74.0)	
Female, n (%)	1122 (51.8)	72 (52.6)	0.92
Symptomatic, n (%)	2120 (97.8)	128 (93.4)	**0.001**
Concurrent PE	532 (24.5)	11 (8.0)	**<0.001**
Provoked, n (%)	1316 (60.7)	105 (75.2)	**<0.001**
**Risk factors**			
Malignancy	291 (13.4)	48 (35.0)	**<0.001**
Catheter-associated	13 (0.6)	61 (44.5)	**<0.001**
Surgical	384 (17.7)	26 (19.0)	0.71
Injury/immobility	572 (26.4)	36 (31.6)	0.98
HRT	18 (0.8)	1 (0.7)	0.90
OCP	67 (3.1)	5 (3.6)	0.71
Chemotherapy	76 (3.5)	26 (18.8)	**<0.001**
Subsequent malignancy	65 (3.0)	2 (1.5)	0.30
**Anticoagulation choice**			
Acute (*p* **< 0.001** overall)			
None	88 (4.1)	1 (0.7)	0.06
Heparin	85 (3.9)	11 (8.0)	**0.022**
LMWH	1435 (66.2)	101 (73.7)	0.06
DOAC	557 (25.7)	20 (14.6)	**<0.001**
Prophylaxis	0 (0)	4 (2.9)	-
Other (danaparoid, heparin, fondaparinux)	3 (0.14)	0 (0)	-
Ongoing (*p* **< 0.001** overall)			
LMWH	309 (14.3)	48 (35.0)	**<0.001**
Warfarin	861 (39.7)	30 (21.9)	**<0.001**
DOAC	868 (40.0)	53 (38.7)	0.70
Other	3 (0.14)	0 (0)	-
None	115 (5.3)	6 (4.4)	0.61
Unknown	12 (0.6)	-	-
**Duration**			
Median for limited (months; IQR)	5 (3.00–7.00)	3 (1.63–6.00)	**0.008**
Lifelong	280	9	-
Unknown	376	17	-
**Complications**			**HR (95% CI),** ***p*-value)**
Major bleeds, n (events/100 PY)	69 (2.09)	6 (5.15)	1.59 (0.69–3.68) *p* = 0.28
Clot progression, n (events/100 PY)	85 (1.18)	7 (6.00)	1.70 (0.78–3.69) *p* = 0.18
Recurrent VTE, n (events/100 PY)	178 (4.12)	10 (4.55)	1.09 (0.57–2.05) *p* = 0.80

Bold values denote statistical significance at the *p* < 0.05 level. Abbreviations: HRT—hormonal replacement therapy; OCP—oral contraceptive pill; LMWH—low molecular weight heparin; DOAC—direct oral anticoagulants; LEDVT—lower extremity deep vein thrombosis; UEDVT—upper extremity deep vein thrombosis; IQR—interquartile range; HR—hazard ratio; 100 PY—100 patient-years.

## Data Availability

The raw data supporting the conclusions of this article will be made available by the authors on request.

## References

[1] Cote L.P., Greenberg S., Caprini J.A., Tafur A., Choi C., Muñoz F.J., Skride A., Valero B., Porras J.A., Ciammaichella M. (2017). Comparisons Between Upper and Lower Extremity Deep Vein Thrombosis: A Review of the RIETE Registry. Clin. Appl. Thromb. Hemost..

[2] Ageno W., Haas S., Weitz J.I., Goldhaber S.Z., Turpie A.G.G., Goto S., Angchaisuksiri P., Nielsen J.D., Kayani G., Pieper K.S. (2019). Characteristics and Management of Patients with Venous Thromboembolism: The GARFIELD-VTE Registry. Thromb. Haemost..

[3] Heil J., Miesbach W., Vogl T., Bechstein W.O., Reinisch A. (2017). Deep Vein Thrombosis of the Upper Extremity. Dtsch. Arztebl. Int..

[4] Lindblad B., Tengborn L., Bergqvist D. (1988). Deep vein thrombosis of the axillary-subclavian veins: Epidemiologic data, effects of different types of treatment and late sequelae. Eur. J. Vasc. Surg..

[5] Thompson R.W. (2012). Comprehensive management of subclavian vein effort thrombosis. Semin. Interv. Radiol..

[6] Lee J.A., Zierler B.K., Zierler R.E. (2012). The risk factors and clinical outcomes of upper extremity deep vein thrombosis. Vasc. Endovasc. Surg..

[7] Drouin L., Pistorius M.A., Lafforgue A., N’Gohou C., Richard A., Connault J., Espitia O. (2019). Épidémiologie des thromboses veineuses des membres supérieurs: Étude rétrospective de 160 thromboses aiguës [Upper-extremity venous thrombosis: A retrospective study about 160 cases]. Rev. Med. Interne.

[8] Tohme S., Vancheswaran A., Mobbs K., Kydd J., Lakhi N. (2021). Predictable Risk Factors of Upper-Extremity Deep Venous Thrombosis in a Level I Trauma Center. Int. J. Gen. Med..

[9] Sajid M.S., Ahmed N., Desai M., Baker D., Hamilton G. (2007). Upper limb deep vein thrombosis: A literature review to streamline the protocol for management. Acta Haematol..

[10] Delluc A., Le Mao R., Tromeur C., Chambry N., Rault-Nagel H., Bressollette L., Mottier D., Couturaud F., Lacut K. (2019). Incidence of upper-extremity deep vein thrombosis in western France: A community-based study. Haematologica.

[11] Baarslag H.J., van Beek E.J., Koopman M.M., Reekers J.A. (2002). Prospective study of color duplex ultrasonography compared with contrast venography in patients suspected of having deep venous thrombosis of the upper extremities. Ann. Intern. Med..

[12] Elman E.E., Kahn S.R. (2006). The post-thrombotic syndrome after upper extremity deep venous thrombosis in adults: A systematic review. Thromb. Res..

[13] Owens C.A., Bui J.T., Knuttinen M.G., Gaba R.C., Carrillo T.C. (2010). Pulmonary embolism from upper extremity deep vein thrombosis and the role of superior vena cava filters: A review of the literature. J. Vasc. Interv. Radiol..

[14] Levy M.M., Albuquerque F., Pfeifer J.D. (2012). Low incidence of pulmonary embolism associated with upper-extremity deep venous thrombosis. Ann. Vasc. Surg..

[15] Prandoni P., Polistena P., Bernardi E., Cogo A., Casara D., Verlato F., Angelini F., Simioni P., Signorini G.P., Benedetti L. (1997). Upper-extremity deep vein thrombosis. Risk factors, diagnosis, and complications. Arch. Intern. Med..

[16] Taylor J.M., Telford R.J., Kinsella D.C., Watkinson A.F., Thompson J.F. (2013). Long-term clinical and functional outcome following treatment for Paget-Schroetter syndrome. Br. J. Surg..

[17] Hoexum F., Jongkind V., Coveliers H.M.E., Wisselink W., Yeung K.K. (2022). Long-Term Outcomes of Nonoperative and Surgical Management of Paget-Schroetter Syndrome. J. Endovasc. Ther..

[18] Kearon C., Akl E.A., Ornelas J., Blaivas A., Jimenez D., Bounameaux H., Huisman M., King C.S., Morris T.A., Sood N. (2016). Antithrombotic Therapy for VTE Disease: CHEST Guideline and Expert Panel Report. Chest.

[19] Bosch F.T.M., Nisio M.D., Büller H.R., van Es N. (2020). Diagnostic and Therapeutic Management of Upper Extremity Deep Vein Thrombosis. J. Clin. Med..

[20] Schulman S., Kearon C., Subcommittee on Control of Anticoagulation of the Scientific and Standardization Committee of the International Society on Thrombosis and Haemostasis (2005). Definition of major bleeding in clinical investigations of antihemostatic medicinal products in non-surgical patients. J. Thromb. Haemost..

[21] Schulman S., Angerås U., Bergqvist D., Eriksson B., Lassen M.R., Fisher W., Subcommittee on Control of Anticoagulation of the Scientific and Standardization Committee of the International Society on Thrombosis and Haemostasis (2010). Definition of major bleeding in clinical investigations of antihemostatic medicinal products in surgical patients. J. Thromb. Haemost..

[22] Lui B., Wee B., Lai J., Khattak Z., Kwok A., Donarelli C., Ho P., Lim H.Y. (2022). A ten-year review of the impact of the transition from warfarin to direct oral anticoagulant—Has venous thromboembolism treatment become safer?. Thromb. Res..

[23] Chopra V., Anand S., Hickner A., Buist M., Rogers M.A.M., Saint S., Flanders S.A. (2013). Risk of venous thromboembolism associated with peripherally inserted central catheters: A systematic review and meta-analysis. Lancet.

[24] Fallouh N., McGuirk H.M., Flanders S.A., Chopra V. (2015). Peripherally Inserted Central Catheter-associated Deep Vein Thrombosis: A Narrative Review. Am. J. Med..

[25] Duijzer D., de Winter M.A., Nijkeuter M., Tuinenburg A.E., Westerink J. (2021). Upper Extremity Deep Vein Thrombosis and Asymptomatic Vein Occlusion in Patients with Transvenous Leads: A Systematic Review and Meta-Analysis. Front. Cardiovasc. Med..

[26] Rosa V., Chaar C.I.O., Espitia O., Otalora S., López-Jiménez L., Ruiz-Sada P., Verhamme P., Muñoz-Torrero J.F.S., Marchena P.J., Monreal M. (2022). A RIETE registry analysis of patients with upper extremity deep vein thrombosis and thoracic outlet syndrome. Thromb. Res..

[27] Al-Samkari H., Connors J.M. (2019). Managing the competing risks of thrombosis, bleeding, and anticoagulation in patients with malignancy. Blood Adv..

[28] Bashir M., Tan S.Z., Jubouri M., Salahia G., Whiston R.J., White R.D., Bailey D.M., Williams I.M. (2022). A narrative review of the surgical management of Paget-Schroetter syndrome: Case series and long-term follow-up. Cardiovasc. Diagn. Ther..

[29] Schneider D.B., Curry T.K., Eichler C.M., Messina L.M., Gordon R.L., Kerlan R.K. (2003). Percutaneous mechanical thrombectomy for the management of venous thoracic outlet syndrome. J. Endovasc. Ther..

[30] Hilleman D.E., Razavi M.K. (2008). Clinical and economic evaluation of the Trellis-8 infusion catheter for deep vein thrombosis. J. Vasc. Interv. Radiol..

